# Short-Term Effects of Exercise Therapy on Muscle Characteristics in Patients With Mild-to-Moderate Amyotrophic Lateral Sclerosis: A Preliminary Case Series Study

**DOI:** 10.7759/cureus.100383

**Published:** 2025-12-29

**Authors:** Naoki Kato, Goichi Hashida, Wataru Sahara

**Affiliations:** 1 Department of Rehabilitation, The University of Osaka Hospital, Osaka, JPN; 2 Department of Orthopedic Surgery, The University of Osaka Graduate School of Medicine, Osaka, JPN

**Keywords:** amyotrophic lateral sclerosis, echo intensity, exercise therapy, muscle thickness, ultrasonography

## Abstract

Background: Exercise therapy is recommended for patients with amyotrophic lateral sclerosis (ALS), but its effects on muscle mass and intramuscular conditions remain unclear. In recent years, ultrasonography has enabled the simple and non-invasive estimation of muscle mass and intramuscular conditions. This study aimed to investigate the short-term effects of exercise therapy on muscle characteristics in patients with mild-to-moderate ALS using ultrasonography.

Methods: Ambulatory patients with ALS and an ALS Functional Rating Scale-Revised (ALSFRS-R) score of ≥30 underwent moderate-intensity exercise therapy for 3-4 weeks. Primary outcome measures included muscle strength (handgrip strength (HGS) and ankle dorsiflexion strength (ADFS)), muscle thickness (MT), echo intensity (EI), and the ratio of muscle strength to muscle thickness for both the forearm muscles and the tibialis anterior muscle. The Bayesian Wilcoxon signed-rank test was used to compare outcomes before and after the intervention.

Results: Six consecutive patients with ALS (median age: 75.5 years; three males (50%) and three females (50%); four with bulbar onset (66.7%) and two with upper limb onset (33.3%)) were included. Following the intervention, the muscle thickness of the forearm muscles and the tibialis anterior muscle decreased. However, qualitative muscle characteristics were partially maintained or improved: the echo intensity of the forearm muscles was maintained, and the ratio of ankle dorsiflexion strength to muscle thickness of the tibialis anterior muscle showed a large increase.

Conclusion: In patients with mild-to-moderate ALS, exercise therapy resulted in short-term qualitative changes in muscle, while a concurrent reduction in muscle mass may have attenuated these effects. The benefits of exercise therapy may have been counteracted by muscle mass loss, suggesting the need for future studies to investigate the combined effects of exercise and nutritional therapy on muscle characteristics and activities of daily living (ADL).

## Introduction

Amyotrophic lateral sclerosis (ALS) is a progressive neurodegenerative disease that develops after middle age and is characterized by the degeneration of motor neurons in the cerebral cortex, brainstem, and spinal cord [1,2]. In Japan, the disease commonly onsets in the 50s-70s, with a reported incidence of 2.2 and a prevalence of 9.9 per 100,000 population [3]. Most patients initially present with muscle weakness in the upper or lower limbs, while some present with bulbar symptoms. The symptoms progress systemically, leading to upper limb dysfunction, gait disturbance, dysarthria, dysphagia, and respiratory failure [1]. The median time from onset to death or the need for ventilatory support is reported to be 32-48 months. Riluzole, edaravone, and high-dose methylcobalamin are approved as drug therapies. However, while these disease-modifying drugs extend survival by a few months, they have not been reported to improve motor function, and their effects are limited [2]. Therefore, the combination of nutritional therapy, respiratory support, and rehabilitation is recommended to maintain or improve activities of daily living (ADL) and quality of life. In patients with bulbar onset, nutritional and respiratory management are prioritized due to the rapid progression of bulbar symptoms, whereas in patients with upper or lower limb onset, the focus is on rehabilitation for limb muscle weakness. However, maintaining residual muscle function is a universal goal of rehabilitation to maintain ADL in all clinical phenotypes [1,2].

In recent years, the effectiveness of exercise therapy for patients with ALS has been demonstrated by meta-analysis [4]. However, the effects of exercise therapy on muscle mass and intramuscular conditions are not well understood. Jensen et al. reported no change in muscle cross-sectional area in patients with ALS after 12 weeks of resistance training [5], but other related reports are limited. The use of ultrasonography to measure muscle thickness (MT) and echo intensity (EI) has enabled the non-invasive estimation of muscle mass and intramuscular conditions, and it has recently been used for monitoring disease progression in patients with ALS [6]. Therefore, we aimed to investigate the short-term effects of exercise therapy on muscle characteristics in patients with mild-to-moderate ALS using ultrasonography.

## Materials and methods

This was a single-center, retrospective case series study reported in accordance with the applicable items of the Strengthening the Reporting of Observational Studies in Epidemiology statement.

Patient selection

The subjects were patients with ALS who were admitted to The University of Osaka Hospital between November 2022 and December 2024 and were referred for rehabilitation. The diagnosis was made by a neurologist based on the Awaji criteria [7]. Inclusion criteria were mild-to-moderate disease, defined as an ALS Functional Rating Scale-Revised (ALSFRS-R) score of ≥30 [4,8], and independent indoor ambulation, without a walking aid, without physical assistance from another person. Exclusion criteria included tracheostomy or mechanical ventilation, severe cognitive impairment, respiratory failure, cardiovascular disease, or malignancy that would affect ADL or the implementation of exercise therapy.

Rehabilitation

The intervention in this study was a comprehensive exercise program conducted by therapists under the direction of a physiatrist. Typically, physical therapy, occupational therapy, and speech-language therapy were prescribed for 40 minutes each on weekdays. However, the prescription of occupational therapy was adjusted at the discretion of the physiatrist; in cases where it was not prescribed, upper limb exercises were integrated into the physical therapy sessions. The intervention period was 3-4 weeks. The main exercises consisted of systemic stepping exercises using a recumbent stepper with simultaneous upper and lower limb drive, and exercises to maintain and strengthen the upper and lower limbs. Strengthening exercises were performed for the lower limbs (e.g., bridging, leg raises, and ankle plantarflexion/dorsiflexion) and upper limbs (e.g., arm raises and gripping), using body weight or weights according to the patient's condition. Approximately half of the physical and occupational therapy session time was dedicated to these exercises, with the intensity adjusted to a moderate level (Modified Borg Scale [9] score of 5 for the limbs). The remaining time was allocated to breathing exercises, range of motion exercises, gait training, and ADL practice. Speech-language therapy included oral motor exercises and voice/articulation practice. Subjective symptoms such as fatigue and shortness of breath were monitored during all sessions. The exercises were paused or discontinued if muscle pain, muscle fatigue, or muscle weakness was observed.

Outcome measures

In general, muscle strength is primarily determined by muscle mass and neural adaptations and is also influenced by the state of non-contractile tissues such as adipose and fibrous tissue, muscle architecture, and stiffness [6,10]. Muscle thickness (MT) is correlated with muscle mass, while echo intensity (EI) is considered an indicator of non-contractile tissue within the muscle. Furthermore, the muscle strength-to-MT ratio is considered a simple measure of specific muscle strength and is reported to be a qualitative indicator of muscle that includes neural adaptations and the state of non-contractile tissue [11,12]. In this study, we measured handgrip strength (HGS) for the upper limbs and ankle dorsiflexion strength (ADFS) for the lower limbs. MT and EI were measured for the forearm muscles (MT-FAMs and EI-FAMs) and the tibialis anterior muscle (MT-TAM and EI-TAM). The muscle strength to MT ratio was also calculated as HGS to MT-FAMs (HGS/MT-FAMs) and ADFS to MT-TAM (ADFS/MT-TAM). HGS was measured using a Grip-D dynamometer (Takei Kiki Kogyo Corp., Niigata, Japan) in a sitting position with the arm hanging down [13]. ADFS was measured with a μ-Tas F2 dynamometer (Anima Corp., Tokyo, Japan) in a supine position with the ankle at 0 degrees of dorsiflexion [14]. For both measures, two maximal contractions were performed, and the average value was used. MT and EI were measured using an Aplio i700 ultrasound system (Canon Medical Systems Corp., Tochigi, Japan). Measurements were taken in a supine position. The MT-FAMs and EI-FAMs were measured at 40% of the proximal forearm length with the shoulder at 0° abduction and the elbow in extension. The MT-TAM and EI-TAM were measured at 25% of the proximal lower leg length with the hip at 0° abduction and the knee in extension [15]. All measurements were performed in B-mode (gain: 85 dB, dynamic range: 70 dB) with an 18 MHz probe, ensuring a 90-degree angle of incidence. For the region of interest, the fascia and bone were excluded, and the largest possible area was analyzed. EI was quantified using ImageJ [16] software on an 8-bit grayscale (0-255). Three images were captured for each measurement, and the average value was used. All ultrasound measurements were performed by a single physical therapist (NK) with 20 years of clinical experience. The muscle strength-to-MT ratio was calculated by dividing the muscle strength value by the MT value. Secondary outcomes included body mass index (BMI), the Barthel Index (BI) [17], and 10-meter maximum walking speed (10mMWS).

Data analysis

Due to the anticipated small sample size, a Bayesian Wilcoxon signed-rank test, which is applicable to small samples, was conducted to compare values at the start and end of rehabilitation. The results are presented as the Bayes factor (BF10) and the posterior median of the standardized effect size (δ) with its 95% Highest Posterior Density Interval (HPDI). BF10 is an index that, unlike a p-value, indicates the extent to which the data support the alternative hypothesis (that a difference exists). A BF10 > 3 was interpreted as evidence for the alternative hypothesis (a difference exists), a BF10 < 0.33 as evidence for the null hypothesis (no difference), and 0.33 ≤ BF10 ≤ 3 as insufficient evidence to draw a clear conclusion about the presence or absence of a difference. Since the prior effect size was unknown, a Cauchy distribution, one of the non-informative prior distributions, was used with a default scale parameter of 0.707 [18]. The sign of the standardized effect size was adjusted to be positive if the value at the end of the intervention was greater than at the start. Effect sizes were interpreted as small (0.2), medium (0.5), or large (≥0.8). To determine practical equivalence, the Region of Practical Equivalence was used with a threshold of δ = ±0.2, with this range considered to have no substantial difference [19,20]. Statistical analysis was performed using JASP version 0.95.0 [21].

Ethical considerations

This study was approved by the Ethics Committee of The University of Osaka Hospital (approval number: 22548). In accordance with the committee's regulations, details of the study were published on the hospital's website (https://www.hosp.med.osaka-u.ac.jp/research/files/rehabilitation7-22548.pdf), and informed consent was waived via an opt-out methodology.

## Results

Six consecutive patients with mild-to-moderate ALS were included; four had bulbar-onset ALS and two had upper limb-onset ALS, and none had lower limb onset. Some eligible patients were discharged within three weeks and were excluded because post-intervention data were unavailable. The individual baseline characteristics of the participants are detailed in Table 1. One patient (16.7%) was unable to have their HGS measured on one side due to severe muscle weakness. In two cases (33.3%), occupational therapy was not prescribed at the physiatrist's discretion; therefore, upper limb exercises were supplemented during physical therapy sessions, with a focus on the recumbent stepper. As a result, their total rehabilitation volume was relatively lower. No adverse events such as muscle pain or muscle weakness were observed during the intervention, and no patients had to stop or reduce the intensity of their exercises.

**Table 1:** Baseline characteristics of participants B: bulbar onset, U: upper limb onset, L: lower limb onset, ALSFRS-R: Amyotrophic Lateral Sclerosis Functional Rating Scale-Revised, %FVC: percent forced vital capacity, GNRI: Geriatric Nutritional Risk Index, Y: yes, S: started during hospitalization, N: no, Ril: riluzole, Eda: edaravone, Resp: respiratory support, Nutr: enteral nutrition

Characteristic	Case 1	Case 2	Case 3	Case 4	Case 5	Case 6
Age (years)/sex	77/female	89/female	61/male	79/male	63/male	74/female
Clinical phenotype	B	B	B	B	U	U
Disease duration (months)	13	8	8	15	33	14
ALSFRS-R	40	41	42	34	33	32
%FVC	61.7	71.9	84.4	44.5	52.3	58.5
GNRI	99.4	95.2	98.5	99.3	93.3	99.1
Medical management	Ril: S, Eda: S, Resp: N, Nutr: N	Ril: S, Eda: S, Resp: N, Nutr: N	Ril: S, Eda: S, Resp: N, Nutr: S	Ril: N, Eda: N, Resp: N, Nutr: N	Ril: Y, Eda: N, Resp: S, Nutr: S	Ril: S, Eda: N, Resp: S, Nutr: S

The results showed that both the MT-FAMs and the MT-TAM decreased from the start to the end of rehabilitation (MT-FAMs: BF10 = 3.54, δ = -0.67; MT-TAM: BF10 = 3.43, δ = -0.64). For the muscle strength-to-MT ratio, no clear conclusion could be drawn for the HGS/MT-FAMs, although a small negative effect was estimated (BF10 = 0.48, δ = -0.27). In contrast, the ADFS/MT-TAM increased from the start to the end of rehabilitation (BF10 = 16.76, δ = 0.93). Regarding EI, there was no change in the EI-FAMs (BF10 = 0.30, δ = 0.07), while for the EI-TAM, no clear conclusion could be drawn, although a small negative effect was estimated (BF10 = 0.47, δ = -0.26). Additionally, BMI decreased (BF10 = 4.75, δ = -1.17), and for 10mMWS, no clear conclusion could be reached, but a small negative effect was estimated (BF10 = 0.49, δ = -0.24). The BI remained unchanged. It is noteworthy that for both MT-FAMs and 10mMWS, the median values increased post-intervention, while the Bayesian analysis indicated a negative effect size. This apparent discrepancy is primarily attributable to the asymmetrical distribution of the data (Table 2).

**Table 2:** Changes in muscle characteristics and activities of daily living Values are presented as median [IQR]. Effect sizes are presented as posterior median [95% Highest Posterior Density Interval]. *BF10 > 3.0: support for alternative hypothesis (difference exists), ΨBF10 < 0.33: support for null hypothesis (no difference) †The median value increased, but the Bayesian effect size was negative due to the asymmetry of the distribution and the reduction in the IQR. BF: Bayes factor, HPDI: Highest Posterior Density Interval, IQR: interquartile range

Item	Baseline	Post-intervention	BF_10_	Effect size (δ) [95% HPDI]
Muscle thickness of forearm muscles (mm)	24.9 [24.3, 27.3]	25.4† [22.6, 25.7]	3.54*	-0.67 [-1.37, -0.05]
Echo intensity of forearm muscles (a.u.)	120.0 [117.8, 130.6]	117.5 [113.5, 129.1]	0.30Ψ	0.07 [-0.44, 0.62]
Handgrip strength-to-muscle thickness of the forearm muscles ratio	0.49 [0.37, 0.54]	0.43 [0.36, 0.54]	0.48	-0.27 [-0.85, 0.27]
Handgrip strength (kg)	12.0 [9.6, 13.8]	10.7 [8.9, 13.1]	0.65	-0.34 [-0.95, 0.21]
Muscle thickness of the tibialis anterior muscle (mm)	16.3 [15.1, 18.8]	14.9 [14.1, 17.4]	3.43*	-0.64 [-1.30, -0.06]
Echo intensity of tibialis anterior muscle (a.u.)	120.1 [113.8, 129.4]	118.3 [111.7, 128.2]	0.47	-0.26 [-0.82, 0.25]
Ankle dorsiflexion strength-to-muscle thickness of tibialis anterior ratio	0.55 [0.49, 0.59]	0.60 [0.59, 0.67]	16.76*	0.93 [0.26, 1.61]
Ankle dorsiflexion strength (kg)	9.4 [8.3, 10.6]	9.7 [8.9, 10.6]	1.20	0.47 [-0.10, 1.07]
Body mass index (kg/m²)	18.4 [18.0, 19.9]	18.1 [17.4, 18.7]	4.75*	-1.17 [-2.90, -0.07]
Barthel Index	90 [70, 98.8]	90 [70, 98.8]	0.54	-0.01 [-1.08, 1.03]
10-meter maximum walking speed (m/s)	0.94 [0.63, 1.38]	1.14† [0.60, 1.52]	0.49	-0.24 [-0.99, 0.44]

## Discussion

This study investigated the effects of exercise therapy on muscle characteristics in patients with mild-to-moderate ALS using ultrasonography. The results suggest that the course of muscle strength, MT, EI, and muscle strength-to-MT ratio varied among measures and potentially differed between the upper and lower limbs.

A decrease in MT over time has been reported in patients with ALS [22]. In this study, both the MT-FAMs and the MT-TAM decreased despite the implementation of exercise therapy. MT is an indicator of muscle mass, and factors contributing to its reduction include muscle atrophy due to motor neuron degeneration, hypermetabolism observed in the early stages after diagnosis, and nutritional deficiencies resulting from dysphagia or dietary restrictions around the time of gastrostomy placement [23]. In this study, all patients had dysphagia, the median BMI was 18.4, and the Geriatric Nutritional Risk Index (GNRI) was 100.8, values close to the lower limit of the normal range (BMI: 18.5, GNRI: 98) [24]. Half of the patients underwent gastrostomy placement during their hospitalization. Furthermore, BMI decreased between the start and end of the intervention, with a large effect size (δ = -1.17). Based on these findings, it was inferred that the influence of nutritional impairment was a major factor in the decrease in MT. This course differs from the report by Jensen et al. [5], where muscle cross-sectional area was maintained before and after exercise therapy. Their study excluded patients with gastrostomy and targeted patients with preserved swallowing function, which may explain the difference from our findings. Thus, we speculate that nutritional impairment was a major factor in the reduction of MT, and muscle mass decreased despite the implementation of exercise therapy. No adverse events, such as muscle pain or muscle weakness, were observed during our intervention. However, in a state of poor nutrition, muscle mass loss can offset the effects of the intervention. This suggests the necessity of monitoring and managing nutritional status in parallel with exercise therapy while avoiding adverse events.

EI in patients with ALS increases as muscle lipomatosis and fibrosis progress with disease advancement [6,22]. In this study, the EI-FAMs were maintained, while the EI-TAM showed a decreasing trend. It has been reported that even short-term exercise therapy can reduce non-contractile tissues such as intramuscular adipose and fibrous tissue, potentially improving echo intensity [10,25]. We also confirmed findings of maintained or decreased echo intensity. It showed a decreasing trend in the lower limbs but was maintained in the upper limbs. A possible reason is that in patients with ALS, the number of remaining motor neurons differs between the site of onset and distal areas, with the rate of motor neuron degeneration and loss being faster at the site of onset [26]. Our subjects had bulbar or upper limb onset, suggesting that the lower limbs had more remaining motor neurons than the upper limbs, which might have made them more responsive to exercise therapy.

There are no reports investigating the course of the muscle strength-to-MT ratio in this disease, but it is assumed that the muscle strength generated per unit of muscle mass decreases as muscle degeneration progresses. In this study, the HGS/MT-FAMs showed a decreasing trend, whereas the ADFS/MT-TAM increased with a large effect size, indicating different courses between the upper and lower limbs. The muscle strength-to-MT ratio is a comprehensive qualitative indicator of muscle, and previous research has reported that it is heavily influenced by neural adaptations [12]. Our subjects had bulbar or upper limb onset, from which it can be inferred that the number of remaining motor neurons was greater in the lower limbs than in the upper limbs. Additionally, the effects of short-term exercise therapy are attributed to neural adaptations such as collateral sprouting and improved motor unit firing and recruitment efficiency [27]. These findings suggest that neural adaptations may have contributed to the increase in the ADFS/MT-TAM. It is suggested that muscle groups with a relatively higher number of remaining motor neurons may have a higher responsiveness to exercise therapy via neural adaptations, making it easier to achieve qualitative muscle improvements. It is also suggested that the muscle strength-to-MT ratio may be a useful auxiliary index for capturing the response to short-term interventions.

The effect sizes for both HGS and ADFS were small. Muscle strength is determined by muscle mass and neural adaptations, and is also influenced by non-contractile tissues. For HGS, MT decreased, but EI and the muscle strength-to-MT ratio were maintained. For ADFS, MT decreased, but EI decreased, and the muscle strength-to-MT ratio increased. In other words, it is conceivable that the decline in muscle strength was suppressed by compensating for the decrease in MT with the maintenance or improvement of qualitative muscle elements such as non-contractile tissue and neural adaptations. The reason why HGS tended to decrease while ADFS tended to improve may be attributed to the difference in the course of the muscle strength-to-MT ratio, which was maintained in the upper limbs but increased significantly in the lower limbs.

In summary, for patients with mild-to-moderate ALS undergoing exercise therapy, our findings suggest that EI can be maintained in the short term, potentially suppressing the worsening of non-contractile tissues such as intramuscular adipose and fibrous tissue. Furthermore, in areas distal to the site of onset (e.g., ankle dorsiflexors), the muscle strength-to-MT ratio showed an improvement (BF10 > 3), suggesting a potential for improving qualitative muscle elements. On the other hand, MT decreased, indicating a reduction in muscle mass. Since muscle strength is primarily determined by muscle mass and neural adaptations, the loss of muscle mass may have weakened the effects of exercise therapy. The main factor for the decrease in MT was presumed to be nutritional impairment. Kunieda et al. reported an increase in muscle strength and mass in a patient with bulbar-onset ALS after five months of nutritional therapy combined with enteral nutrition and exercise therapy [28]. Further investigation is needed to clarify the effects of long-term exercise therapy combined with nutritional management on muscle characteristics and ADL improvement.

Regarding the study design, limitations include the small sample size, the heterogeneity of the study population (i.e., the inclusion of both bulbar and upper limb-onset patients), the lack of a control group, and the lack of standardized nutritional management, in addition to the study being conducted at a single institution with only Japanese subjects and the short intervention period. As this was part of clinical care, although we attempted to standardize exercise intensity and unify exercise content through physical therapy, the composition of exercises and the presence or absence of occupational therapy could be confounding factors. Large-scale comparative studies and long-term observational studies are needed in the future. Regarding drug therapy, one patient was taking riluzole, and four patients started taking riluzole or receiving edaravone during hospitalization. Although these drugs are said to have little effect on improving muscle strength [2], their impact on muscle characteristics is unknown and requires further investigation. Furthermore, our subjects did not include patients with lower limb onset, resulting in a biased clinical phenotype. In lower limb onset, swallowing function is relatively preserved, so the impact of nutritional impairment, considered the main cause of MT reduction, is smaller. However, patients with lower limb onset often have lower ADL and difficulty walking from the time of diagnosis compared to other phenotypes [29]. Therefore, patients with bulbar or upper limb onset are more often candidates for active exercise, and we believe our sample reflects one aspect of the clinical setting. Finally, the intervention did not lead to improvements in ADL or walking ability. This study was short-term and had a small sample, so we could not draw a clear conclusion about the impact on ADL. Future verification studies with a control group are needed to examine the relationship with clinical outcomes in more detail.

## Conclusions

This study investigated the short-term effects of exercise therapy on muscle characteristics in patients with mild-to-moderate ALS using ultrasonography. The results suggest that EI was maintained, potentially suppressing the worsening of non-contractile tissue. In areas distal to the site of onset, the muscle strength-to-MT ratio improved, suggesting a potential for improving qualitative muscle elements. On the other hand, MT decreased, and it is speculated that the effects of exercise therapy may have been counteracted by a reduction in muscle mass. Nevertheless, our findings demonstrate that muscles still have the potential to respond positively to exercise despite disease progression. Maintaining muscle quality is likely a key factor in preventing functional decline in ADL. Further investigation is needed into the effects of long-term exercise therapy combined with nutritional management on muscle characteristics and improvement in ADL, as nutritional impairment may have been a contributing factor.

## Appendices

The Awaji criteria [7] and ALSFRS-R [8] are used with permission.
